# Synthetic gene regulation for independent external induction of the *Saccharomyces cerevisiae* pseudohyphal growth phenotype

**DOI:** 10.1038/s42003-017-0008-0

**Published:** 2018-01-22

**Authors:** Georgios Pothoulakis, Tom Ellis

**Affiliations:** 1 Centre for Synthetic Biology and Innovation, Imperial College London, South Kensington Campus, Exhibition Rd, London, SW7 2AZ UK; 2 Department of Bioengineering, Imperial College London, South Kensington Campus, Exhibition Rd, London, SW7 2AZ UK

## Abstract

Pseudohyphal growth is a multicellular phenotype naturally performed by wild budding yeast cells in response to stress. Unicellular yeast cells undergo gross changes in their gene regulation and elongate to form branched filament structures consisting of connected cells. Here, we construct synthetic gene regulation systems to enable external induction of pseudohyphal growth in *Saccharomyces cerevisiae*. By controlling the expression of the natural *PHD1* and *FLO8* genes we are able to trigger pseudohyphal growth in both diploid and haploid yeast, even in different types of rich media. Using this system, we also investigate how members of the *BUD* gene family control filamentation in haploid cells. Finally, we employ a synthetic genetic timer network to control pseudohyphal growth and further explore the reversibility of differentiation. Our work demonstrates that synthetic regulation can exert control over a complex multigene phenotype and offers opportunities for rationally modifying the resulting multicellular structure.

## Abstract (web summary)

Georgios Pothoulakis and Tom Ellis report the construction of a synthetic gene regulation system for inducing pseudohyphal growth in *Saccharomyces cerevisiae*. This multicellular yeast phenotype can now be switched on and off via external control in a variety of conditions.

## Introduction

As biological research and efforts in synthetic biology accelerate the incorporation of ever-more complex systems into organisms, they are also shifting from tasks performed in individual cells to tasks achieved by more complex multicellular systems such as pattern formation. Formation of multicellular patterns is a widely observed characteristic found in a large number of organisms and is the result of intricate arrangements of differentiated cells under the control of development and differentiation mechanisms. These arrangements enable the formation of structures that serve specialist roles, such as tissues and organs^1^.

While multicellular phenotypes are expressed commonly by multicellular organisms, they can also emerge in organisms traditionally considered unicellular. A classic example are biofilms, which are microbial cell aggregations that secrete extracellular polymeric substances forming a protective matrix around a colony population^2^. Unicellular organisms offer the advantage of being reliable and easy to genetically manipulate, while also not naturally displaying multicellular phenotypes in rich growth media. One such organism is *Saccharomyces cerevisiae* yeast, which can display a variety of characteristics normally associated with multicellularity, specialization, and pattern formation. *S. cerevisiae* is able to take multicellular growth forms such as flocs, flors, biofilms, and filaments in response to environmental condition by changing the way that the cells adhere to each other and to biotic or abiotic substrates using proteins called adhesins or flocculins^3,4^.

Filamentation is an important differentiation mechanism for many fungal species as a response to external stimuli such as nitrogen starvation while, for some yeast species like *Candida albicans*, the phenotype it is also connected to pathogenicity^5,6^. There is great variety in the morphology among the different filamenting fungal species and in the case of *S. cerevisiae* yeast cells are forming pseudohyphae: chains of elongated cells fully-separated by cytokinesis but attached through adhesion proteins^5^. Pseudohyphal growth is a highly complex phenotype that involves a large number of processes including substrate adhesion, bud site selection and cell morphogenesis^7^. Both haploid and diploid yeast cells create kinds of pseudohyphae formation as a stress response although the underlying genetic and morphological differences between the two cell types during the expression of this response leads to a distinction. The term pseudohyphal growth is usually reserved for diploid cells, while the term invasive growth is used for haploid cells due to the characteristic invasion of the colonies into agar substrate^5^. Extensive research has elucidated many of the genetic and biochemical mechanisms behind pseudohyphal growth, a complex multi-faceted phenotype regulated by four highly characterized signaling pathways—the Ras2/cAMP-PKA pathway, the protein kinase Snf1 pathway, the TOR (target of rapamycin) pathway and the MAPK pathway^5,8^. Interestingly, only diploid *MAT*a/*MATα* yeast strains can form pseudohyphae, as pseudohyphal growth is a characteristic of the bipolar budding pattern. However, haploid cells lacking *BUD4*, which encodes a protein responsible for the conversion of the bipolar budding pattern to an axial budding pattern, are known to grow following the bipolar budding pattern and so offer potential to enable filament formation from haploid cells^9^. While pseudohyphal growth is in general a natural response to nutrient starvation, i.e., nitrogen stress or glucose deprivation, it can also be induced independently by manipulating the expression of specific gene targets^10^. Two such targets are the genes *PHD1* and the *FLO8*, which were previously investigated based on their ability to induce pseudohyphal growth when overexpressed in diploid yeast^10,11^.

In previous work, we showed that the simplest multicellular phenotype that is normally triggered by stress in *S. cerevisiae*—flocculation—can be put under the control of synthetic gene networks in standard lab strains^12^. Here we take this a step further, using synthetic gene networks introduced into the genome of S288C-derived yeast in order to enable the more complex pseudohyphal growth multicellular phenotype to be externally triggered in rich growth media in both haploid and diploid strains. This is achieved by using synthetic genetic regulation to control the filamentous growth master regulator genes and in two different designs, we describe versions that work in galactose-based media and glucose-based media with different chemical inducers. By monitoring the growth characteristics directed by these networks in strains with *BUD* gene deletions, we are able to observe the roles of *BUD8* and *BUD9* in defining the multicellular growth patterns. Finally, we explore the applicability of established yeast synthetic network designs in the context of growth phenotype control by using multigene networks to control the transition back from pseudohyphal to normal colony growth over a set amount of time^12^. This work demonstrates the use of synthetic regulation to control and reverse a complex multigene phenotype in yeast that directs a multicellular growth morphology from normally unicellular cells.

## Results

### A synthetic gene network for inducible pseudohyphal growth

**Fig. 1:**
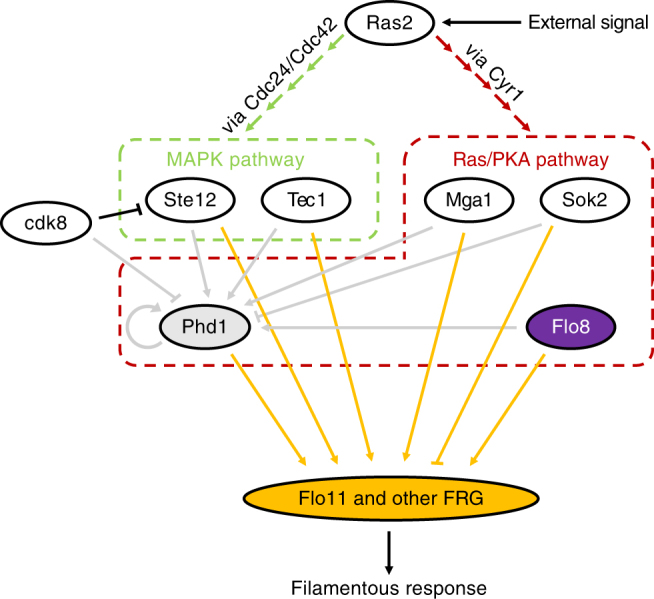
Regulation of Muc1/Flo11 protein. External signals such as nitrogen starvation activate Ras2, which in turn triggers both MAP kinase and Ras/PKA pathways through a cascade involving Cdc24/Cdc42 and Cyr1, respectively^8^. Several factors from both pathways have been shown to control Phd1 expression including Ste12 and Tec1 of the MAP kinase pathway and Mga1, Sok2, and Flo8 from the Ras/PKA pathway and Phd1 itself^11^. In addition, Cdk8 which is expressed in normally growing cells has been shown to cause Phd1 and Ste12 protein destabilization^11^. Ultimately, several filamentous response genes (FRG) including *Flo11* are regulated by Phd1, Flo8, Ste12, Tec1, Mga1, and Sok2 and direct the phenotype. Arrows indicate upregulation while lines with bars indicate inhibition^5, 11^

To construct a synthetic gene network to independently induce filamentation in rich media we chose to target the *PHD1* and *FLO8* master regulator genes. Both the Phd1 and Flo8 proteins are involved in the pseudohyphal growth cascade of the cAMP-PKA pathway and act as transcriptional regulators of the *MUC1/FLO11* gene that encodes for a flocculin that plays a central role in pseudohyphal growth and in other yeast multicellular phenotypes (Fig. 1)^5,13^. The *MUC1/FLO11* gene is regulated by a large promoter bound by multiple transcription factors from several signaling pathways and its expression can have dramatic effects on cell adherence with other substrates^5^. In nutrient rich conditions, many of these transcription factors, including Phd1, are down-regulated thus prohibiting pseudohyphal growth^11^. Past studies have shown that overexpression of *PHD1* induces pseudohyphal growth in Σ1278b diploid strains even in nutrient-rich conditions, although deletion of this gene appears to have no negative effect in filamentation^8,10^. Gimeno and Fink have also shown that over-expressing *PHD1* in specific *BUD4* negative Σ1278b-based haploid strains causes the pseudohyphal growth pattern during nutrient starvation^10^. However, even in nitrogen-limiting conditions, expression of pseudohyphal growth is impossible without proper expression of the *FLO8* gene^11,14^.

Most commonly-used lab and industrial yeasts such as those derived from the S288C strain carry a mutation in the *FLO8* gene that leads to the expression of a truncated version of the protein (flo8-1) making pseudohyphal growth impossible^14–16^. This mutation is not present in the Σ1278b strain used to study yeast multicellular phenotypes. Removing this mutation to enable proper Flo8 protein expression in S288C cells restores its filamentation capabilities^14^. Raithatha et al.^11^ have shown that co-expression of the un-truncated version of the *FLO8* gene with a version of the *PHD1* gene that carries a natural polymorphism on the 92nd codon (S92F) found in the Σ1278b strain, enhances filamentation of the S288C strain. This polymorphism eliminates a Cdk8-dependent phosphorylation site, thus further stabilizing the Phd1 protein.

**Fig. 2:**
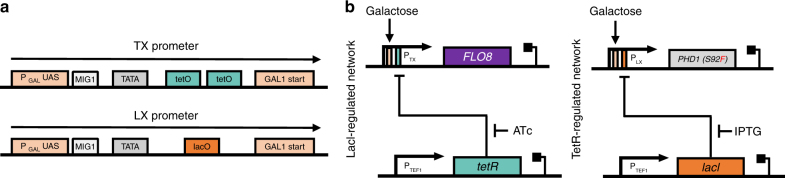
Designing synthetic regulatory networks for pseudohyphal growth induction. **a** Diagrams of the TX and LX synthetic promoters. Both promoters contain the GAL1 upstream activating sequence (UAS), which is required for galactose activation but is normally repressed in glucose, a MIG1 protein repressor recognition site that inhibits activation in glucose, a TATA box and either *tetO2* or *lacO* operator sites. Each promoter is represented by a black arrow containing all critical sites shown as colored rectangles. Transcription from each promoter occurs at the defined ‘start’ sites. **b** Diagram of the synthetic gene networks for controlling pseudohyphal growth. *PHD1(S92F)* is under the control of the GAL1-based LX promoter carrying a Lac operator (lacO) site and induced by IPTG, while *FLO8* is under the control of the GAL1-based TX promoter carrying a Tet operator (tetO2) site and controlled by ATc. Both *lacI* and *tetR* genes are expressed from the constitutive *TEF1* promoter. Genes are shown as colored boxes, promoters as arrows. Regulation is shown as rectangles inside the promoters (orange for LAC, green for TET, and pink for GAL1)

Two previously described synthetic promoters were used to generate an initial synthetic regulation network that triggers pseudohyphal growth when yeast are grown in galactose media and given external chemical inducers (Fig. 2a). The two promoters used, TX and LX, are repressible by the bacterial TetR and LacI repressors, respectively, when these transcription factors are co-expressed^12,17^. Promoter repression from the TetR and LacI proteins can be relieved by the addition of anhydrotetracycline (ATc) and Isopropyl *β*-D-1-thiogalactopyranoside (IPTG), respectively^12^. Since both TX and LX promoters are based on GAL1, a natural yeast promoter repressed in the presence of glucose and induced by galactose, both promoters are only active in galactose-containing media^18,19^. And since both the *tetR* and *lacI* genes originate from *E. coli*, for inhibition to occur both need to be added to the constructs^20,21^. Thus, combining the TX and LX promoters with genomically integrated cassettes that constitutively express *tetR* and *lacI*, results in the complete synthetic gene network. Initially, a network with two genes under independent regulation was constructed, so that the growth morphology of cells overexpressing the modified *PHD1* gene (S92F mutant) and the fully-functioning *FLO8* gene could be independently assessed (Fig. 2b).

As pseudohyphal growth is a characteristic of the bipolar budding pattern normally only observed in diploid cells, these two gene networks were first incorporated into the BY4743 diploid yeast strain. Rather than making any modifications to the natural, inactivated *PHD1* and *FLO8* genomic loci in BY4743, the synthetic gene networks were integrated at the well-characterized *URA3* landing-pad locus elsewhere in the genome. Since we also desired to test these networks in haploid cells we repeated integration of the constructs as in the diploid but now in a BY4741 haploid with a *bud4* gene knockout (strain Y02569). Knockout of this gene is known to force the haploid cells to grow in a bipolar budding pattern and thus may aid in establishing pseudohyphal growth^22^.

**Fig. 3:**
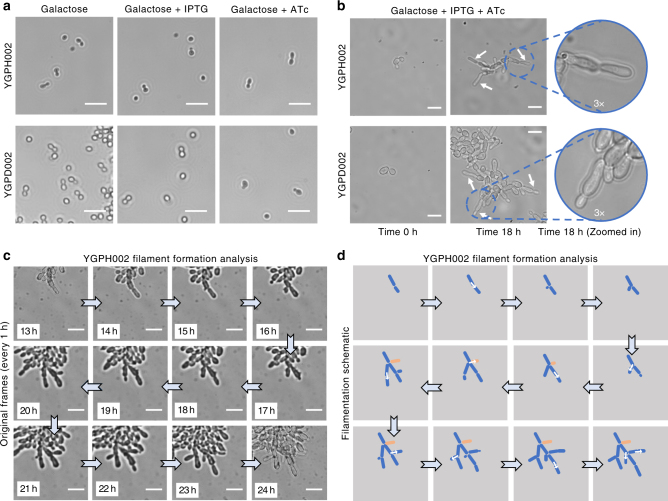
Implementing inducible synthetic regulatory networks for pseudohyphal growth induction. **a** Inverted microscope images of haploid YGPH002 and diploid YGPD002 strains in different induction conditions. Images were taken after 24 h of growth in galactose media or galactose media supplemented with either 1 mM IPTG or 250 ng/ml ATc. Brightfield images were acquired using a 20× objective. Small white lines are scales which correspond to 20 μm. **b** Images of the haploid YGPH002 and diploid YGPD002 strains taken at time 0 h or after 18 h of growth inside the ONIX microfluidic platform in galactose media with 250 ng/ml of ATc and 1 mM IPTG supplemented. Areas of the 18 h time point frames have been enlarged by 3× to highlight filament formation. Brightfield images were acquired using a 60× objective. White arrows are pointing to newly formed filaments. Small white lines are scales which correspond to 10 μm. **c** Time-lapse microscopy and (**d**) filament formation analysis of the haploid YGPH002 strain when pseudohyphal growth is induced. Cells were induced for 24 h. Here, frames from 1 h intervals starting from the 13 h time point were selected and magnified in order to highlight the generation of branched formations from cells following a unipolar pattern. White arrows highlight growth of new branches from mother cells. Brightfield images were taken using a 60× objective. The white lines correspond to scales with a length of 10 μm. The cell shown in orange is not clearly visible after the 8th frame since it’s covered by other cells

The two resulting strains, YGPH002 (haploid) and YGPD002 (diploid), were first assessed for their ability to grow in filamentous form in induction conditions. In the absence of either or both inducer chemicals in galactose growth media, no filamentous growth is seen (Fig. 3a). This demonstrates that overexpression of just one of the two genes (*PHD1(S92F)* or *FLO8*) is not enough to induce pseudohyphal growth. However, both strains shown signs of flocculation when grown in galactose media, even in the absence of IPTG and ATc. This is likely indicative of some leaky expression from the synthetic networks possibly as a result imperfect repression of the TetR and LacI-regulated promoters and cells being sensitive to even the smallest amounts of the Phd1 or Flo8 proteins. As Flo8 is directly involved in the flocculation phenotype it is most likely leaky expression of this gene that leads to a degree of flocculation.

In galactose media with both ATc and IPTG inducers present, both the haploid and diploid trains showed clear morphological differences, visibly forming filaments as they grow (Fig. 3b). This filamentous growth was imaged on the ONIX microfluidics platform with images of cells taken using an inverted microscope at 0 and 18 h post induction. Cells appeared considerably elongated and attached to their mother cells, validating the anticipated phenotype.

Following verification of the desired phenotype, we next generated a time-lapse to track the haploid YGPH002 cells as they transition from normal to pseudohyphal growth and generate filaments (Fig. 3c, d). The results confirmed that upon induction haploid cells form filaments that branch off as new daughter cells appear. They also confirmed that upon pseudohyphal growth induction, cells turn from a bipolar to a unipolar budding pattern where new buds form on the pole opposite of the birth scar in an effort to branch away from the center of the colony. A further observation that affirms successful induction of pseudohyphal growth is the visible “waiting” of the mother cells to further divide while their daughter cells are elongating. It is clear that daughter buds appear only after the previous daughter cells have reached maximum size. It was proposed by Kron et al.^23^ that during pseudohyphal growth, mitosis in the mother cell is delayed by a G2 checkpoint while the daughter elongates, and this results in synchronization of budding between mother and daughter cells, something not seen during normal budding growth.

The above results verify that it is possible to design and construct synthetic regulatory networks capable of inducing the pseudohyphal growth phenotype and subsequent filament formation in *S. cerevisiae*, even when these cells are not stressed by external factors. Importantly, this work shows that the synthetic networks can induce filamentation in haploid lab yeast.

### Inducible pseudohyphal growth independent of carbon source

The initial two synthetic gene networks enable stressor-free externally inducible pseudohyphal growth in haploid and diploid *S. cerevisiae*, however this system is only functional in galactose-rich growth media. Therefore, we next turned to the previously developed Z3EV-mediated gene induction system described by McIsaac et al.^24^ to obtain external induction in the absence of galactose in the media. Z3EV is an artificial transcription factor which is a fusion of the mouse Zif268 DNA binding domain, the ligand binding domain of the human estrogen receptor and VP16 (viral protein 16). In the presence of *β*-estradiol this selectively binds to promoters containing Zif268 binding motifs and activates their expression^25^.

**Fig. 4:**
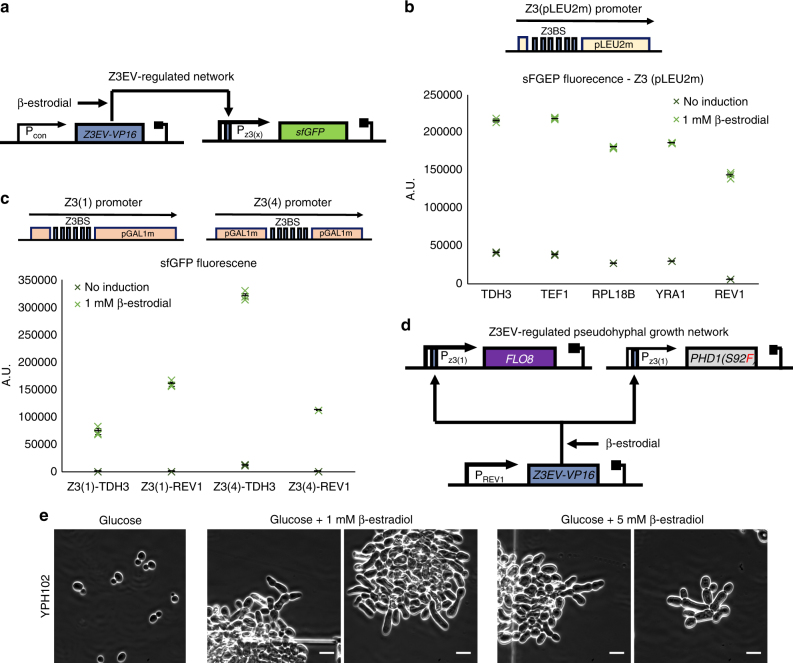
Extending external inducible pseudohyphal growth to glucose-rich media. **a** Diagram of the Z3 promoter characterization system. The *yEGFP* fluorescent marker is under the control of one of the three Z3 promoter derivatives, activated by the *Z3EV-VP16* transcription factor in the presence of β-estradiol. *Z3EV-VP16* is expressed from a constitutive promoter. Genes are shown as colored boxes, promoters as arrows. *Z3EV-VP16* regulation sites are shown as blue rectangles within the promoters. **b** Characterization of the Z3(pLEU2) promoter when paired with different combinations of constitutive promoters driving *Z3EV-VP16* expression (TDH3, TEF1, RPL18B, YRA1, or REV1). The yEGFP fluorescent output of five different constructs is tested when induced with 1 mM of β-estradiol for 5 h. Data shows fluorescence values from three repeats (colored X markers) as determined by flow cytometry, black bars indicate mean values, error bars indicate standard error. **c** Characterization of the Z3(1) and Z3(4) promoters when paired with either the TDH3 or REV1 constitutive promoters driving *Z3EV-VP16* expression. The green fluorescence output of the four different constructs is tested when induced with 1 mM of β-estradiol for 5 h. Data shows fluorescence values from three repeats (colored X markers) as determined by flow cytometry, black bars indicate mean fluorescence values, error bars indicate standard error. **d** Diagram of the final Z3EV-regulated pseudohyphal growth network. Both the *FLO8* and *PHD1(S92F)* genes are under the control of the Z3(1) promoter while *Z3EV-VP16* is expressed from the constitutive REV1 promoter. Genes are shown as colored boxes while promoters as arrows. *Z3EV-VP16* regulation sites are shown as blue rectangles within promoters. **e** Inverted microscope images of the haploid YPH102 strain taken after 28 h of growth inside the ONIX microfluidic platform in glucose media without β-estradiol or with either 1 or 5 mM of β-estradiol supplemented. Brightfield images were acquired using a 60× objective. Small white lines are scales and correspond to 10 μm

Following the approach described by McIsaac et al., we constructed our own Z3EV-activated promoter by placing 6 Zif268 binding sites (from now on referred to as Z3BS) upstream of a minimal LEU2 promoter to create promoter Z3(pLEU2m) (Fig. 4a). In previous uses, the Z3EV transcription factor has been constitutively expressed from the strong ADH1 promoter, however as we required promoter leakiness to be minimized, we opted to reduce Z3EV expression to the minimum required for good expression^25^. Thus, we created a library of constructs carrying five constitutive promoters of various strengths driving Z3EV expression. To characterize these, the Z3(pLEU2m) promoter was placed upstream of a *sfGFP* gene. In the presence of the β-estradiol inducer, the Z3EV transcription factor binds to the Z3(pLEU2) promoter and activates *sfGFP* transcription which can be quantified by measuring the green fluorescence of cells using flow cytometry.

All five constructs were transformed into Y02569 haploid yeast cells and induced with 1 mM of β-estradiol for 5 h and measured compared to their uninduced counterparts (Fig. 4b). The mean fluorescence values with the five different promoters for Z3EV expression reveal that the relative activation levels of the Z3(pLEU2m) promoter are comparable. This therefore allows us to eliminate the use of strong expression for this transcription factor. Furthermore, there is a notable fold-increase in expression upon induction in all cases and this is most prominent for the REV1 promoter, the weakest of those tested here. The construct with this promoter, has the most desirable on/off characteristics needed for the synthetic gene network but still shows some leaky expression from the Z3(pLEU2m) promoter when no inducer is present.

In order to improve on this, we replaced the Z3(pLEU2m) promoter with two designs containing the GAL1 core promoter that more closely match the P1 and P4 promoters described by McIsaac et al. These two new promoters, named Z3(1) and Z3(4) were tested as before in cells where Z3EV expression is driven by the strongest (TDH1) and weakest (REV1) of the five constitutive promoters. Performance of their expression as determined by flow cytometry, is shown in Fig. 4c. The previously observed leaky expression from both TDH1 and REV1 promoters has now been drastically decreased, and in particular for the REV1-containing constructs the leakage is practically eliminated.

Using these optimized promoter combinations, a final design with Z3EV expression from the REV1 promoter and *PHD1(S92F)* and *FLO8* expression from the Z3(1) promoter was selected for induction of pseudohyphal growth in glucose-containing media (Fig. 4d). This synthetic regulatory network, designed to be dependent only on the presence of β-estradiol, was introduced into haploid Y02569 cells to create strain YGPH102. This strain was then grown in glucose, and induced with varying concentrations of β-estradiol for 28 h while under observation in the ONIX microfluidics platform (Fig. 4e). The resulting images show that cell morphology indeed changes considerably due to the induction of pseudohyphal growth with external inducer, with cells elongating and visibly staying attached to each other in the growing colony. In contrast when no inducer is added, cells follow normal growth patterns.

### Altering the inducible pseudohyphal morphology in haploids

We next asked whether growth morphology could be further perturbed through programmed genetic changes. We focused on the *BUD* gene family that is known to affect budding patterns during growth by controlling the cell sites where new buds appear during division and are also known to be involved in regulating the filamentous growth MAPK pathway^26,27^. Two proteins in particular play major roles in bud site selection in bipolar budding diploid cells; Bud8 and Bud9. The Bud8 protein localizes primarily to the distal pole, forcing distal pole site selection, while the Bud9 protein localizes primarily to the proximal pole to give the reverse outcome^28^. Previously, it has been shown that creating gene knockouts of the *BUD8* gene in diploid cells forces cells to bud almost exclusively from the proximal pole, while knocking out the *BUD9* gene forces cells to bud almost exclusively from the distal pole^29^. In addition, Harkins et al.^28^ has shown that similar results can be taken by controlling the relative levels of expression of the *BUD8* and *BUD9* genes rather than via gene deletion. In normal haploid cells, deletion of these genes does not have any effects on the axial budding pattern and does not affect cell viability or growth rates, although they do play a role during haploid invasive growth where the Bud8 protein promotes this phenotype while the Bud9 impedes it^28,30^. Therefore, we chose to investigate the knockout of these two genes and their effects on morphology using our inducible pseudohyphal growth haploid strain.

**Fig. 5:**
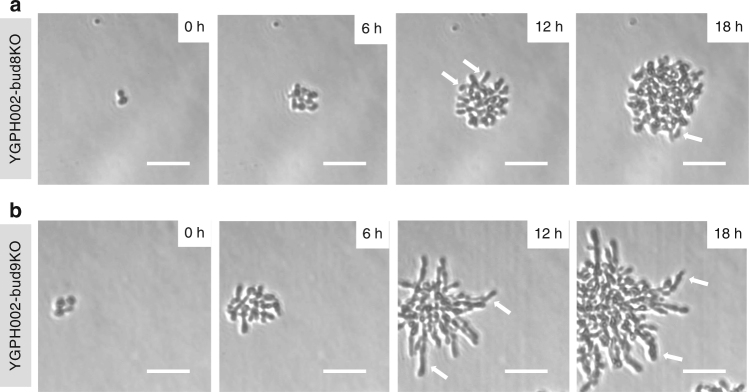
Induced pseudohyphal growth in haploid cells with *BUD8* or *BUD9* gene deletion. **a** YGPH002-bud8KO time-lapse. **b** YGPH002-bud9KO time-lapse. Arrows highlight some of the filaments formed as a result of the induced pseudohyphal growth phenotype. Cells are grown for 18 h on 1.2% agarose pads made with 2% synthetic complete galactose media, supplemented with 400 ng/ml ATc and 1 mM IPTG. Brightfield images were captured using a 20× objective and a Phase 1 contrast filter every 10 min but only selected frames are shown here. White arrows point to cells exhibiting filamentous-like morphology. White lines represent scale bars with a length of 50 μm

The *BUD8* and *BUD9* genes were individually deleted from our haploid YGPH002 strain, in each case by transforming and recombining the linear *K. lactis URA3* marker into the relevant chromosomal loci to disrupt the gene. In both cases, many viable colonies were seen post transformation, confirming that knockouts of these genes do not affect cell viability^28,31^. The two new strains were externally induced as before and their pseudohyphal growth capabilities were assessed using an inverted microscope and growth in galactose media. Cells were grown on agarose pads to enable generation of time-lapse images (Fig. 5, Supplementary Movies 1 and 2). These images clearly show that the -*bud8* strain (YGPH002-bud8KO) has difficulty creating filaments that extend outwards from the colony, although elongated cells are seen. Instead, the colony appears to be concentrated at the center with new buds developing inwards, a strong indication of proximal budding. In contrast, the -*bud9* strain (YGPH002-bud9KO), clearly exhibits a classic pseudohyphal growth phenotype with extensive filaments forming and always extending outwards of the center of the colony.

### Synthetic regulation for phenotype reversal during growth

The orthogonal, non-native transcription factors used within our inducible synthetic gene networks allow the switch to pseudohyphal growth to be connected to inputs other than external induction. In the absence of inducer chemicals, filamentous growth can be activated by any regulation that triggers Z3EV expression (in the glucose media system) or that inhibits expression of LacI and TetR (in the galactose media system). This design theoretically enables the change in growth phenotype to be linked to a wide variety of inputs that change gene expression in yeast, such a light-based induction, cell-to-cell signaling and environmental sensing^32–34^. It also enables the growth phenotype to be connected to existing synthetic gene networks that have been developed previously in work by the yeast synthetic biology field^35^.

To demonstrate this feature, we modified our galactose-media pseudohyphal growth induction system to link it to a synthetic gene network motif previously described as a genetic ‘timer’. This motif, consisting of mutual inhibition of LacI and TetR, delays the return of an initial gene expression state for several generations after an initial pulse of chemical induction, in this case with ATc^12^. It enables a population to grow performing strong expression of one or more genes for multiple generations, before switching these off and reverting back to expression of other genes as before the induction. When linked to *FLO8* and *PHD1* expression, the timer motif is designed initiate a population to differentiate and grow for several generations in pseudohyphal mode before the cells within this then begin reverse their differentiation and revert back to normal budding unicellular growth. This can be used to create a population on a plate that initially forms expansive filaments, before then budding to form a full colony.

**Fig. 6:**
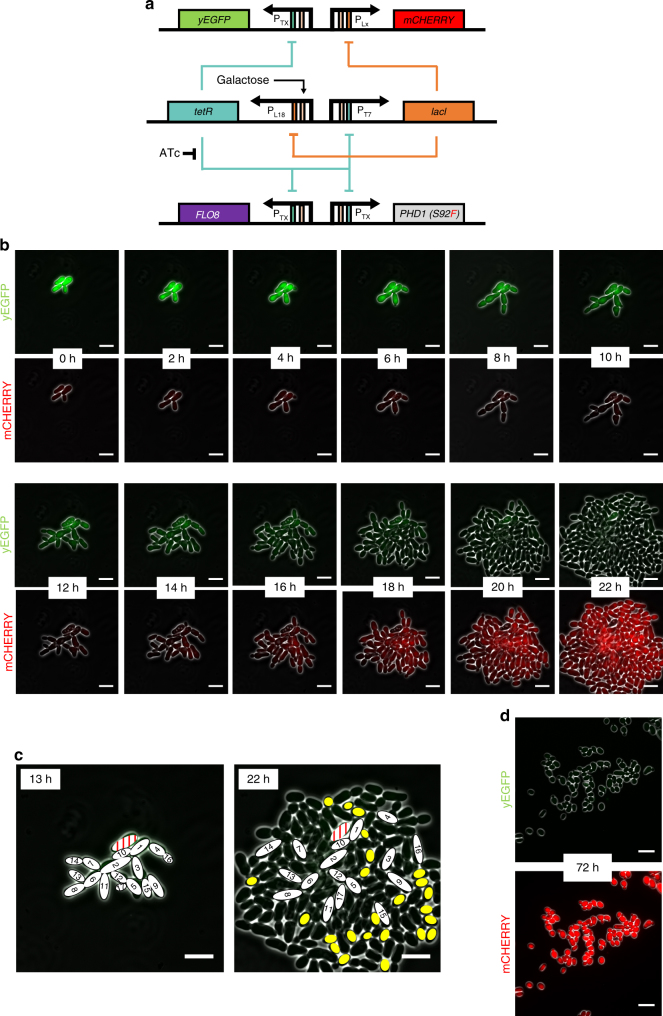
Using a genetic timer network to temporally control pseudohyphal growth within a growing colony. **a** Diagram of the T7-L18 synthetic timer network interfaced with expression of pseudohyphal growth genes and green and red fluorescent proteins. The *lacI*, *PHD1*, *FLO8*, and *yEGFP* genes are under the control of the TX promoter or its derivative, T7. The *mCHERRY* gene is expressed from the LX promoter while its derivative L18 promoter is controlling the *tetR* gene. Genes are shown as colored boxes, promoters as arrows. Regulation sites are shown as rectangles inside promoters (orange for lac, green for tet, and pink for galactose). **b** Time-lapse fluorescence microscopy of the YGPTIMER strain carrying the T7-L18 genetic timer. Cells pre-treated with ATc for 24 h were grown inside the ONIX microfluidics platform in synthetic complete galactose media after ATc had been removed by washing. Brightfield, green (yEGFP) and red (mCherry) fluorescence images are taken every 10 min. Here, selected frames from 2 h intervals are shown. **c** Analysis of cell position and shape for the YGPTIMER strain carrying the T7-L18 genetic timer. The exact same cells present at 13 h after the removal of ATc are shown in the final colony image at 22 h after ATc removal. Cells are numbered based on the order of appearance. One cell that arrests after emerging and never gives a daughter cell appears in red. Yellow ovals represent cells that appear oval after reaching full growth and are also detached from their mother cells. **d** Inverted microscope images of the YGPTIMERX strain taken after 72 h of growth in synthetic complete galactose media after ATc had been removed by washing. The green (top) and red (bottom) fluorescence channels are each combined with the brightfield channel to enhance cell visibility. Images are taken using the 60× objective and the Phase 3 contrast filter. White lines represent scale bars with a length of 10 μm

For the genetic timer motif (Fig. 6a) we modified the previously described T7-L18 genetic timer which uses mutated versions of the TX and LX promoters with reduced maximum expressions to ensure a long reset time after initial induction is removed^12^. Since the *LACI* gene originates from *E. coli*, we encoded a strong c-Myc nuclear localization signal (NLS, amino acid sequence PAAKRVKLD) into the C-terminal of the protein to ensure this regulator gave strong repression in *S. cerevisiae*^36^. Both the *PHD1(S92F)* and *FLO8* genes necessary for pseudohyphal growth induction were placed downstream of TX promoters making them co-regulated by the TetR levels in the yeast. By default, the T7-L18 timer rests at the *tetR* ON state and so in this configuration, the filamentation genes are repressed when cells grow normally in galactose-rich media. Upon induction with ATc, *tetR* regulation is supressed, the network switches to the *lacI* ON state (pseudohyphal growth) and stays there as long as the inducer is present. After removal of the inducer the network slowly reverts back to the *tetR* ON state at a rate dependent on the relative strengths of the opposing T7 and L18 promoters. For characterization purposes, this synthetic gene network also included two further genes, the *yEGFP* and *mCHERRY* genes that encode green and red fluorescent proteins, respectively. These were expressed from further copies of the TX and LX promoters and used to monitor transition of the timer motifs from one state to another in a manner similar to that used before by Wu et al.^37^.

The complete six gene network was constructed as three cassettes and integrated into the haploid Y02569 strain to create a strain called YGPTIMER (Fig. 6a, Supplementary Movies 3 and 4). Cells from this strain were then pre-treated with ATc to induce filamentation and then washed to remove this inducer before being transferred into the ONIX microfluidics platform for live-cell imaging over 22 h (Fig. 6b). The initial cells of the colony grew in filamentous form to expand from the center for the first 12 h. During this time, they exhibited a strong yEGFP signal associated with low levels of TetR and high levels of LacI in each cell. Between 12 and 18 h this signal decreased and mCherry expression began, signifying a switch to lower LacI levels, back to the *tetR*-ON state of the timers. Accompanying this, the number of cells in the colony now begins to increase more rapidly and outward exploration with new filaments starts to cease. By 22 h most of the new cells within the colony are now red in fluorescence rather than green, and exhibit a more oval morphology compared to the initial filamenting cells, suggesting that budding unicellular growth starts appearing in the colony. To get a better understanding on how the colony behaves after the initial induction of filamentation and the resulting cell placement when the system has fully reset, the localization of the filamenting cells from the 13 h and 22 h time points was analyzed (Fig. 6c). This revealed that the cells that had been undergoing pseudohyphal growth in the first 13 h still individually looked filamentous after 22 h, but were now no longer adhered to one another, instead being broken apart by the rapid growth of the new budding cells. Many of new cells in the later timepoints are oval shaped and virtually all cells on the outer edge of the colony show detachment from their mother cells. Yet there is also a clear lag between the switching of fluorescent protein production from green to red and the disappearance of the various features of pseudohyphal growth. As the phenotype is reversed, we first see increased occurrence of detachment and then the new cells gradually grow less elongated, although not all new cells can be said to be oval. During the experiment, all cells continue to bud exclusively from the distal pole (a sign of unipolarity) suggesting that this is the slowest differentiation feature to revert. Finally, to confirm the full reversion of the system, we imaged a timer strain left to grow in liquid phase for 72 h after removal of the inducer and observed cells with standard budding morphology exhibiting high red fluorescence and no green fluorescence (Fig. 6d).

## Discussion

This work utilized synthetic gene regulatory networks to activate yeast pseudohyphal growth even in nitrogen rich media. Initially two separate inducible synthetic regulatory networks based on the TetR and LacI-regulated TX and LX promoters, respectively, were shown to work in both haploid and diploid cells that grow in galactose media (Fig. 3). To our understanding, this is the first time where pseudohyphal growth has been induced in a haploid strain in rich media. Previously, *PHD1* overexpression has been shown to induce pseudohyphal growth in *bud4* mutant haploid cells only in nitrogen limited (SLAD) media^10^. In addition, it has been shown that a constitutive pseudohyphal growth phenotype is observed in *elm1Δ fus3Δ* haploid cells^38^. Importantly, both strains formed filaments without the presence of external stressors. A similar design chosen to be independent of the carbon source was implemented with Z3-based regulation and upon induction of this the haploid strain showed altered growth morphology but with a certain number of cells remaining spherical, and the overall filamentation characteristics appearing less strong than in galactose-grown strains (Fig. 4). A likely explanation for this is that the Z3(1) promoter is weaker than the TX and LX promoters and thus upon induction leads to lower *PHD1* and *FLO8* expression. Taken together this work highlights the use of promoter-based synthetic regulatory networks for controlling complex phenotypes. As the regulated promoters used are modular and orthogonal it is possible that these networks could be implemented in other species. Indeed, related TetR-regulated promoters have been used for gene expression control in many organisms, including *Candida albicans*^12,39^.

Enabling control over the induction of the pseudohyphal growth in cells grown in glucose and nitrogen-rich conditions potentially leads to filaments with different metabolic behavior from cells that traditionally form filaments under nitrogen starvation or glucose deprivation. Central metabolic functions in particular, are very sensitive to glucose availability and there is a general decrease in cytoplasmic ribosomal mRNA availability when there is very low glucose availability^40^. In addition, the absence of glucose can lead to general inhibition of translation in *S. cerevisiae* and also acts as a trigger of invasive growth in wild-type haploid strains that follow the axial budding pattern^41,42^. The limited biomass formation caused by filamentation in combination with normal glucose-enabled metabolism could potentially be a route towards higher yields of biochemical products from metabolically engineered cells that also have the morphology control system described here^43^.

Previously it has been proposed that mutations in the *BUD8* gene eliminate pseudohyphal growth and based on our experiments of triggering *PHD1* and *FLO8* overexpression when Bud8 is absent, that does appear to be the case and cells do exhibit a proximal pole bias (Fig. 5)^7^. Having said that, cell morphology appears to change and elongated cells become apparent. Since bud site selection is affected by the relative levels of the Bud8 and Bud9 proteins, further exploration could be achieved through coordinated overexpression or repression of these two genes rather than via direct gene knockouts. This could enable additional possibilities for rational synthetic regulation of colony morphology, colony size and the pattern and directions of multicellular growth.

An important goal of synthetic regulatory network design is the ability to construct and implement predictable and mathematically-described systems that are able to achieve complex phenotypical results and here that was attempted by linking our synthetic pseudohyphal switch to a previously-described timer network (Fig. 6). The intentional slow-reversion of the phenotype in this experiment yields insights into how the cells are behaving when their morphology is under the control of synthetic mutual inhibition network. Overall, we observe an initial expansion of the area of the colony due to pseudohyphal growth but at the cost of slow cell growth. Eventually, as newly-formed cells start switching to a more unicellular growth mode and appearing less elongated, growth rate accelerates and filament formation brakes as a result of both mechanical forces of newly formed cells that push old ones and elimination of adhesion. The unexpected speed with which the unicellular-growing yeast takes over the colony is not surprising as they can divide much faster in rich media. Less obvious is why the genetic timer system used here only enforced the pseudohyphal phenotype for 4 or so generations over the first 20 h. When previously described in liquid growth experiments, the T7-L18 timer used here took five times as long to reset back after initial induction^12^. It is clear that there are major differences in the intracellular environment and gene expression in filamenting cells compared to in unicellular yeast.

Overall, this work describes how synthetic gene networks can be used to control the growth of *S. cerevisiae* yeast into filamented multicellular colonies, both in haploid and diploid standard lab strains and in rich growth media too. Bottom-up engineered synthetic multicellularity from unicellular yeast or using other organisms offers opportunities for exciting applications of synthetic gene networks, where populations of cells can be repurposed for new tasks (e.g., to grow patterned materials) or can be used to investigate the principles of natural pattern formation (e.g., the roles of cell-to-cell signaling, feedback and regulation). Control over pseudohyphal growth as described here represents a major initial step towards achieving predicable multicellular patterned growth from unicellular cells. With further genetic engineering, it could presumably be possible to gain even greater control of the subsequent colony morphology. Synthetic gene networks could be employed to provide additional control over budding localization along filaments, and differential gene expression through the colony (e.g., altered expression of genes in cells at the edge of the filaments vs. those in the center of the colony). These networks could employ quorum sensing mechanisms, cell-to-cell signaling or mother- or daughter-cell specific promoters in order to have differential regulation between the cells. Theoretically, genetic programs could be written that define when and where fractal-like filamentous growth patterns occur and how ‘branched’ the filaments are (by controlling budding frequency and location). These synthetic multicellular patterns could be utilized in scenarios where increased surface area and substrate removal from a colony of yeast is beneficial.

Ultimately, this work aims to accelerate the use of synthetic biology in the exploration and generation of genetically-encoded multicellular pattern formation programs, and therefore it is especially valuable that this work has been achieved in one of the most widely-used model organisms and works in standard lab growth conditions. As such this work offers a toolkit towards programming synthetic multicellular morphologies within growing 2-dimensional yeast colonies.

## Methods

### Strains and cultures

All engineered strains we derived either from the haploid *S. cerevisiae* Y02569 (BY4741; *MAT*a; *ura3*Δ*0*; *leu2*Δ*0*; *his3*Δ*1*; *met15*Δ*0*; YJR092w::*kanMX4*) strain provided by EUROSCARF or from the diploid *S. cerevisiae* BY4743 (*MAT*a/α; *his3*Δ*1*/*his3*Δ*1*; *leu2*Δ*0*/*leu2*Δ*0*; *LYS2*/*lys2*Δ*0*; *met15*Δ*0*/*MET15*; *ura3*Δ*0*/*ura3*Δ*0*) strain, also provided by EUROSCARF. The YGPH002 and YGPD002 strains were created by transforming both the pGPY002 and pGPY003 plasmids into the Y02569 and BY4743 strains respectively. The YGPH078, YGPH079, YGPH080, YGPH081, YGPH082, YGPH092, YGPH093, YGPH094, and YGPH095 strains were created by transforming the corresponding pGPY078-82 and pGPY092-95 plasmids into the Y02569 strain. The YGPH102 strain was created by transforming the pGPY102 plasmid into the Y02569 strain. The YGPH002-bud8KO and YGPH002-bud9KO knockout haploid strains were created through the insertion of the *K. lactis URA3* gene in place of the *BUD8* or *BUD9* gene respectively thus replacing the corresponding open reading frame (ORF) in its entirety and part of the upstream promoter sequence in YGPH002 cells. The YGPTIMER and YGPTIMERX strains were created by subsequently transforming the pGPY006, pT7L18 and pGPY062 plasmids in the Y02569 strain. An NLS sequence (amino acid sequence PAAKRVKLD) was added to YGPTIMER through CRISPR editing. Cells are cultured in synthetic complete drop-out glucose media (SC-Glu) for transformation and general proliferation and in synthetic complete drop-out galactose media (SC-Gal) media with either IPTG, ATc or both supplemented. During the time-lapse and ONIX experiments, cells were grown at 30 °C inside the microscope. For all other cultures, cells were grown in liquid cultures at 30 °C with shaking at 225 rpm.

### Plasmid construction

The pGPY002 plasmid carrying the *PHD1(S92F)* gene under the LX promoter and the *lacI* gene under the TEF1 promoter and the pGPY003 plasmid carrying the *FLO8* gene under the TX promoter and the *tetR* gene under the TEF1 promoter were based on the pLVGI and pTVGI plasmids respectively (both based on pRS4D1 plasmid) described in Ellis et al.^12^. The *PHD1* and *flo8-1* genes were acquired from the YPH500 yeast strain through colony PCRs that also introduce restriction sites compatible with the pLVGI and pTVGI plasmids thus replacing the *yEGFP* gene. The *PHD1* gene inside the pGPY002 plasmid was altered on the 92^nd^ codon so that it encodes Phenylalaline (TTT codon) instead of Serine (TCT codon) while the *flo8-1* gene of the pGPY003 plasmid was altered to remove the early stop codon (TAG-142^nd^ codon) and replace it with TGG so that it creates the fully functional *FLO8* gene. To make the new plasmids compatible with the Y02569 and BY4743 strains, the *TRP1* markers of both the LVGI and TVGI plasmids were replaced with the *HIS3* and *LEU2* markers respectively taken from pRS based plasmids through simple restriction digestion and ligation. For the Z3 promoter experiments all plasmids were assembled using the MoClo method of modular assembly in combination with the Yeast ToolKit created by the Dueber lab^44^. All necessary promoter, open reading frame (ORF) and terminator parts were amplified accordingly and stored in parts plasmids compatible with the rest of the YTK kit. The Z3BS, Z3(1), Z3(4), and Z3EV parts were created using sequences obtained from the pRS416-yZ3EV-Z3pr-yEGFP (RB3579) which was a gift from David Botstein (Addgene plasmid # 69100). From there, cassette plasmids containing the whole genes of interest were created and finally assembled together using the pYTK096 *URA3* integrating plasmid as a backbone thus generating the pGPY078-82, pGPY092-95 and pGPY102 plasmids. The pGPY006 plasmid was based on the pGPY002 plasmid. The LX promoter of the *PHD1(S92F)* gene and the *lacI* gene along with the TEF1 promoter were removed completely and replaced with a TX promoter and the *FLO8* gene controlled by TX as well. The pT7L18 plasmid carrying the *tetR* and *lacI* genes controlled by the L18 and T7 promoters respectively is described in Ellis et al.^12^. The pGPY062 plasmid carrying the *yEGFP* gene under the TX promoter and the *mCHERRY* gene under the LX promoter is a modified version of the p714 plasmid described in the same study with its *TRP* marker replaced by *URA3* to make it compatible with the Y02569 strain^12^. Supplementary Data 1 contains sequences of all plasmids used in this study in commented Genbank format.

### Linear BUD gene family replacing fragment generation

In order to generate the linear fragments that enabled the creation of the *BUD8* and *BUD9* knockouts the *K. lactis URA3* gene was PCR amplified from the pMirage plasmid provided by Dr Ben Blount (Imperial College London) using three different primer pairs that introduced the necessary overlaps for homologous recombination to directly replace the *BUD8* or *BUD9* genes at their genomic loci and a part of their upstream regions.

### Inverted microscope image capture

Images were taken through 20x/0.45 or 60x/1.40 CPI60 objectives mounted on a Nikon Eclipse Ti inverted microscope with live cells imaged on plain slides, 1.2% SC-Gal agarose pad slides or using the ONIX microfluidic platform. To visualize the samples a Phase filter 1 or 3 is used to enhance contrast and Brightfield illumination. For fluorescence capture, excitation, emission filters, and exposures were respectively 480 nm, 535 nm, 1000 ms for the GFP channel (yEGFP) and 532 nm, 590 nm, 2000 ms for Cy3 channel (mCherry). During time-lapse experiments, the software autofocus function of the microscope is used to adjust for any potential movement of the cells during growth in order to keep clear track of the samples. The microscope experiment of Fig. 2b, the time-lapse experiment of Fig. 2c and the time-lapse experiment of Fig. 3c, d were carried out using the CellASIC ONIX Microfluidic platform (Merck Millipore). For cell growth, in most cases flow rate was adjusted between Psi = 2 and Psi = 4 which was determined to be good for yeast cells. For haploid yeast cells the Y04C-02-5PK plates were used. The time-lapse microscopy experiments shown on Fig. 3 were carried out on 1.2% SC-Gal agarose pad slides including 400 ng/ml ATc and 1 mM IPTG which were prepared following a protocol adapted from Rines et al.,^45^. NIS-Elements Microscope Imaging Software (Nikon) is used for capturing and ImageJ (National Institutes of Health) is used for image presentation.

### Flow cytometry analysis

Flow cytometry assays were performed using the Attune NxT flow cytometer with the Attune NxT autosampler attachment from ThermoFisher Scientific. A 488 nm laser was used for excitation of green fluorescence detecting through a 530 nm band-pass filter (BL1). The voltages of the FSC, SSC and BL1 channels for the promoter characterization experiments were 200, 320 and 480 respectively. A threshold of 3.0 × 10^3^ A.U. was applied to the forward (FSC) scatter to minimize non yeast events. Data analysis was performed using FlowJo software (Tree Star), gating samples for forward scatter and side scatter to exclude non-yeast events and obtaining fluorescence values from BL1-H (height) channels.

### Data availability

Time-lapse movies of the experiments shown in Figs. 5a, b and 6a, b are provided in Supplementary Movies 1–4. High resolution versions of all microscopy images shown in the manuscript are available via Figshare at DOI: 10.6084/m9.figshare.5660179^46^. Maps of new plasmids generated in this study are provided in Supplementary Data 1 in commented GenBank format and all plasmids will be made available via Addgene. All data generated or analyzed during this study that are not included in this published article and its Supporting Information files are available upon request.

## Electronic supplementary material

Description of Additional Supplementary Files

Supplementary Movie 1

Supplementary Movie 2

Supplementary Movie 3

Supplementary Movie 4

Supplementary Data 1
