# Supplementary material for: Synthetic gene regulation for independent external induction of the Saccharomyces cerevisiae pseudohyphal growth phenotype
Source: Commun Biol. 2018 Jan 22;1:7. doi: 10.1038/s42003-017-0008-0 (PMC6123699; doi:10.1038/s42003-017-0008-0)
Supplement: Supplementary file 1 — Description of Additional Supplementary Files [file 42003_2017_8_MOESM1_ESM.docx]

**Description of Additional Supplementary Files**

File Name: Supplementary Movie 1

Description: YGPH002-bud8KO time-lapse

File Name: Supplementary Movie 2

Description: YGPH002-bud9KO time-lapse

File Name: Supplementary Movie 3

Description: Time-lapse fluorescence microscopy of the YGPTIMER strain carrying the T7- L18 genetic timer (GFP/BF channels)

File Name: Supplementary Movie 4

Description: Time-lapse fluorescence microscopy of the YGPTIMER strain carrying the T7-

L18 genetic timer (mCHERRY/BF channels)

File Name: Supplementary Data 1

Description: Plasmid maps in commented Genbank format
